# Effects of Steam Explosion on Curcumin Extraction from Fresh Turmeric Chips

**DOI:** 10.3390/plants13233417

**Published:** 2024-12-05

**Authors:** Umnat Imcharoen, Pornchai Rachtanapun, Parichat Thipchai, Ruangvate Sae Eng, Sinchai Chinvorarat, Petch Jearanaisilawong

**Affiliations:** 1 Department of Mechanical and Aerospace Engineering, King Mongkut’s University of Technology, North Bangkok, Bangkok 10800, Thailand; s6301001910521@email.kmutnb.ac.th (U.I.); sinchai.c@eng.kmutnb.ac.th (S.C.); 2 Division of Packaging Technology, School of Agro-Industry, Faculty of Agro-Industry, Chiang Mai University, Chiang Mai 50100, Thailand; pornchai.r@cmu.ac.th; 3 Center of Excellence in Agro Bio-Circular-Green Industry (Agro BCG), Chiang Mai University, Chiang Mai 50200, Thailand; 4 Nanoscience and Nanotechnology, Faculty of Science, Chiang Mai University, Chiang Mai 50200, Thailand; parichat.t245@gmail.com; 5 World Premier Packaging Co., Ltd., Bangkok 10160, Thailand; worldpack218@gmail.com

**Keywords:** turmeric chips, disintegrated, abruptly, efficient process, dissipated energy per mass

## Abstract

The purpose of this research is to study the effect of the steam explosion (SE) process on curcumin extraction from fresh turmeric chips. Fresh turmeric chips abruptly disintegrated during the steam explosion process. The investigation into the turmeric particles following the steam explosion process in the SEM micrographs revealed that the formation of surface cracks and cavities led to an increase in the surface area of turmeric particles. Curcumin extracted from turmeric particles after the steam explosion process yielded 3.24% (*w*/*w*), which was comparable to the yield of 3.98% (*w*/*w*) from finely ground turmeric particles, while the steam explosion used 74% less energy than the grinding process. Therefore, the steam explosion process is an efficient process compared to untreated and conventional mechanical grinding methods. On average, the turmeric particles decreased in size when the dissipated energy per mass increased. The curcumin yield from the steam explosion exhibited a linear positive correlation with the dissipated energy per mass. FTIR, TG/DTG, and DSC analyses on the turmeric particles after the steam explosion process showed that the compounds exhibited no change in chemical structure, higher thermal decomposition properties, and higher purity, respectively. The results of this research can be applied to find optimal conditions for extracting curcumin and predicting the yield of curcumin. Additionally, they can be applied to evaluate the process condition in commercial applications.

## 1. Introduction

Turmeric is native to countries in South and Southeast Asia. It is mostly grown in Malaysia, Pakistan, China, and India. Turmeric (*Curcuma longa* L., Zingiberaceae) can be cultivated in all regions of Thailand [1]. People have used turmeric as a spice, preservative, food coloring, and an ingredient in cosmetics and dyes. Furthermore, turmeric possesses therapeutic qualities and has been used for its medicinal effect. Turmeric comprises two primary active compounds, volatile oil and curcuminoids, which are yellow-orange compounds that consist of curcumin, demethoxycurcumin, and bisdemethoxycurcumin. Curcumin is insoluble in water but soluble in organic solvents such as hexane, methanol, and ethanol. The maximum quantity of curcuminoids in ethanol is soluble at 9.15% by weight, which requires less time to dissolve and yields more quantity than when dissolved in methanol [2]. Numerous medical benefits of curcumin have been documented, including anti-inflammatory [3,4], immunomodulatory [5], antioxidant [6], hypolipidaemic [7], antimicrobial [8], anticarcinogenic [9], antitumor [10,11,12], radioprotective [13], neuroprotective [14], hepato-protective [15], nephroprotective [16], and cardio-protective activities [17]. Furthermore, it inhibits the growth and accelerates the death of colon cancer cells [18].

Machine grinding is one of the mechanical cell-disintegration methods used in the curcumin-extraction process. Although it does not require sophisticated equipment and high extraction yield, the cost of production is high because of the solvent and energy consumption [19,20]. High-pressure assisted extraction pretreatment is an environmentally friendly technology for curcumin extraction because the process uses less energy and has a minimum effect on the molecules of curcumin. The disadvantages are the high cost of the instrument and the batch processing [21]. Using a pulsed electric field to aid in curcumin extraction is one method of the electroporation treatment approach. High extraction yield, quick process, and low energy consumption are the benefits. The high cost of instruments and the need to eliminate air bubbles for consistent electric field distribution are the drawbacks [21]. A fast, simple, and economical method to extract curcumin is microwave assistance. Pectin and starch in turmeric may diffuse into the solvent when the cell is destroyed, which could lead to thermal degradation [22]. Curcumin extraction using ultrasound-assisted extraction has proven to be a rapid, intense, and efficient method. The drawbacks are that the material must be small to achieve a homogenous effect and that thermal degradation reduces the curcumin content. When the cell is destroyed, pectin and starch in the turmeric may diffuse to the solvent [23]. The process of ohmic heating-assisted extraction is fast, uses the minimum amount of organic solvent, and is particularly useful in laboratory settings. The high starch content in turmeric, which may encourage gelatinization and inhibit curcumin extraction, and the high cost of the equipment are the drawbacks [24]. Enzyme-assisted extraction has minimal usage of solvents, and the extraction is rapid and efficient, but the enzymes are expensive, and curcumin is unstable in water [25]. Additional heat treatment at 90 °C for 48 h degraded the curcuminoid and reduced its antioxidant qualities, whereas the sample kept at room temperature for the same period showed no changes [26].

Another technique that combines mechanical and thermal methods is the steam explosion process, which is a low-cost and energy-efficient innovation. It is used in the size reduction of wood process, is environmentally friendly, and utilizes 70% less energy than the fine grinding method [27]. It has been extensively used for pretreatment procedures for breaking down fibers and destroying cell walls in a number of processes, including phytochemicals, bioactives, and extraction. The steam explosion process was used in the extraction of flavonoids from sumac fruits, and the results showed that this process yielded a higher amount of flavonoid than grinding of the raw material [28]. The maximum flavonoid concentration was produced by steam explosion pretreatment of seabuckthorn pomace under optimal conditions, which included 2 MPa steam pressure, 88 s in time, and 60 mesh particle size. This was 246% greater than the technique that did not involve steam explosion [29]. High extraction findings of phenolic compounds were obtained by steam explosion pretreatment of chrysanthemum morifolium ramat cv. Hangbaiju mesh size 250 microns [30]. After steam explosion pretreatment (0.2 MPa and 3 min), flavonoid yield extracted from fig leaf was increased by 55.9% compared with that from untreated fig leaf [31]. After barley bran was steam-exploded for 120 s at 220 °C, the yield of total soluble phenol content was nine times higher than that of untreated samples, and the antioxidant capacity was increased [32]. However, the steam explosion pretreatment on turmeric chips has not been investigated.

This research aimed to study the effects of the steam explosion process on fresh turmeric chips. Scanning electron microscopy (SEM) was used to examine the cell structure of turmeric rhizomes and particles after steam explosion, and a laser particle size distribution analyzer (PSD) was used to examine the distribution of particle sizes. The ethanol solubility method was used to extract the curcumin, and HPLC was used to determine its content. The energy consumption of the steam explosion and fine grinding methods were also compared. The relationship between the dissipated energy per mass and the extracted curcumin yield was established for the estimation of curcumin in the steam explosion process. We examined the effects of the chemical structure of turmeric particles by FTIR, the thermal properties by DSC, and the thermal decomposition properties by TG/DTG in different processes in comparison with the curcumin product.

## 2. Materials and Methods

### 2.1. Materials

**Figure 1:**
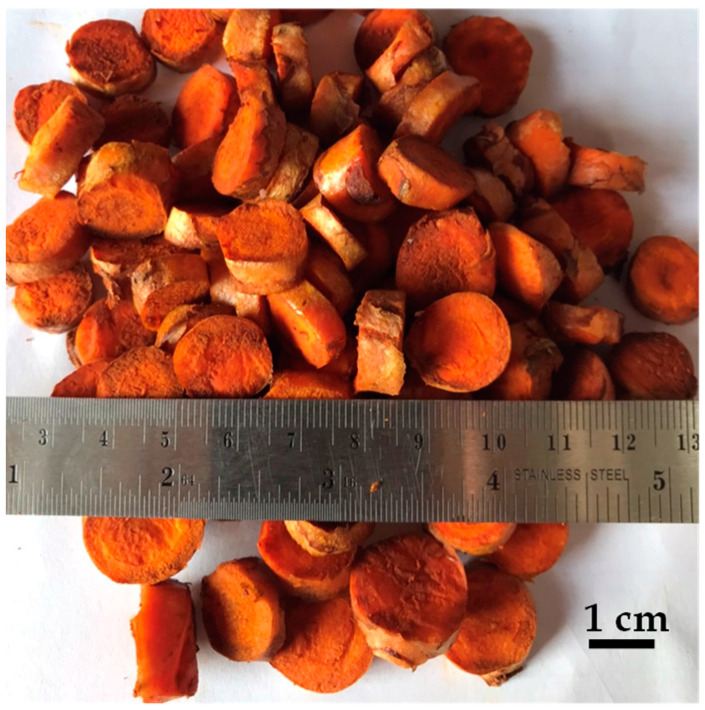
Dimensions of fresh turmeric chips.

The rhizomes of turmeric (*Curcuma longa* Linn., Zingiberaceae) were harvested at the age of 18 months from the planting area, Khao Wong Subdistrict, Ban Ta Khun District, Surat Thani Province, southern Thailand. This planting area had yields of curcuminoids of 8.41 ± 0.04% *w*/*w* [1]. The raw material was prepared by cleaning and washing the fresh rhizomes of turmeric, then chopping them into chips without peeling. As illustrated in Figure 1, the averaged diameter is 16 ± 1 mm, the averaged thickness is 6 ± 1 mm, wet base moisture is 87.13%, bulk density is 486.81 kg/m^3^, true density is 1023.56 kg/m^3^, and porosity is 55.44%. The experimental pretreatments of turmeric fresh chips were performed at pressure ranging from 10 to 25 barG. After steam explosion pretreatment, turmeric particles were filtered with a 105 μm nylon filter bag, dried with a centrifuge, and air dried. The dried turmeric chips had a wet base moisture content of 7.52%, a bulk density of 452.38 kg/m^3^, a true density of 1049.38 kg/m^3^, and a porosity of 56.89%. After being pulverized in the Thomas Model 4 Wiley mill, the dried turmeric particles were sieved through a 60 mesh screen and collected on an 80 mesh sieve. Curcumin was extracted by using 99.9% ethanol (QRëC, Wellington, New Zealand) and compared to the standard curcumin (Merck, Darmstadt, Germany).

### 2.2. Morphological Analysis

A scanning electron microscope (SEM, QUANTA 450, FEI Co., Ltd., Hellsboro, OR, USA) was used to analyze the morphology of the turmeric rhizome and powders. Samples of the longitudinal and transverse cross sections of the turmeric rhizome were mounted with resin, polished with fine 1000-grit sandpaper, and coated with gold for 30 s. The 80 mesh finely crushed turmeric powders and dried turmeric particles after the steam explosion at 240 °C and 4 min residence time were employed for surface analysis. High vacuum mode, 10 kV voltage, and magnifications of 1000× and 5000× were used for the observation.

### 2.3. Steam Eplosion Pretreatment

#### 2.3.1. Steam Explosion Machine

**Figure 2:**
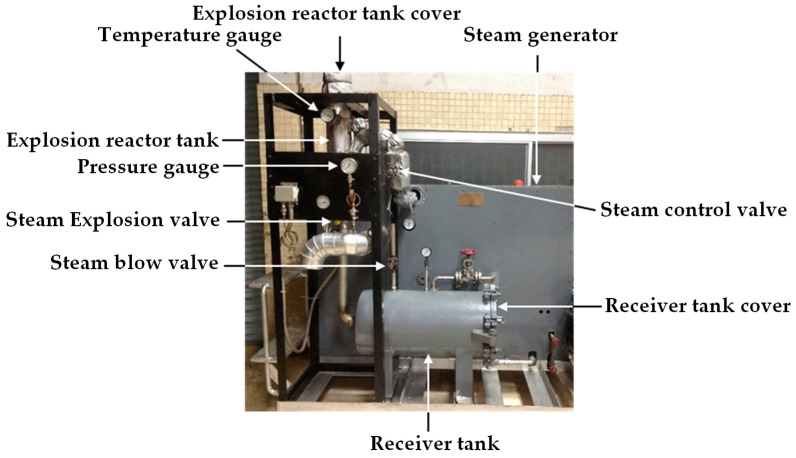
The batch steam explosion machine.

The steam explosion set intended for use with a steam generator is the batch steam explosion depicted in Figure 2. The 1.5 L explosion reactor tank is made such that the cover can be opened to place turmeric chips on top. Using the steam control valve from the steam generator, the amount of steam can be regulated to enter the explosive reactor from 0 to 30 barG. A temperature gauge and a pressure gauge are used to measure the temperature and steam pressure inside the explosive reactor. The ball valve with a diameter of 36.83 mm is used as a steam explosion valve. When turmeric chips are cooked by steam inside the reactor for a specified period, the steam explosion valve is activated, and the chips flow through the explosion valve into the receiver tank. Then, the turmeric chips will disintegrate and spread inside the receiver tank. The blow valve is opened to release the steam inside the receiver tank. As steam is blown through the steam blow valve, a portion of steam condenses inside the receiver tank and mixes with turmeric particles. The turmeric samples are collected by opening the receiver tank cover, and the samples are swept out of the receiver tank with a plastic spatula onto the tray.

#### 2.3.2. Modeling of Disintegration of Fresh Turmeric Chips

**Figure 3:**
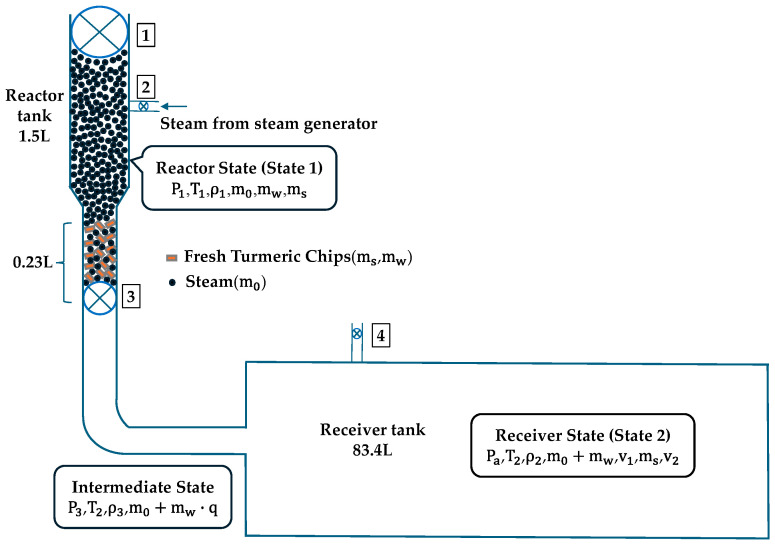
Schematic diagram of steam explosion machine valve: (1) sample charging valve, (2) saturated steam supply valve, (3) steam explosion valve, (4) blow valve.

The receiver tank and reactor tank have a ratio of 55.6 in the steam explosion machine design. The volume percentage in the reactor tank for the experiment involving the explosion of 100 g/batch of fresh turmeric chips is 15.3%, as depicted in Figure 3. The 100 g sample rests in a 50 mm-diameter pipe that allows for sudden flow through the explosion valve upon opening. This pipe and valve design result in a sample volume of 0.23 L.

The initial state parameters (State 1) of the steam inside the explosion reactor tank are represented by the following parameters: $P_{1}$, $T_{1}$, $\rho_{1}$, $m_{0}$, $m_{w}$, and $m_{s}$, where $P_{1}$ is the steam pressure; $T_{1}$ denotes the steam temperature; $\rho_{1}$ denotes the steam density; $m_{0}$ denotes the mass of steam; $m_{w}$ denotes the mass of water inside the fresh turmeric chips and $m_{s}$ denotes the mass of dry turmeric chips. The instantaneous explosion state parameters (State 2) inside the receiver tank are $P_{a}$, $T_{2}$, $\rho_{2}$, $m_{0}+m_{w}$, $v_{1}$, $m_{s}$, and $v_{2}$. $P_{a}$ is the pressure inside the receiver tank that is equivalent to atmospheric pressure. $T_{2}$ is the temperature at which steam is produced when water in fresh turmeric chips evaporates $\rho_{2}$ is the density of steam from fresh turmeric chips, and the velocities of the steam and turmeric particles are represented by $v_{1}$, $v_{2}$, respectively. The state parameters between the initial state and the inside receiver tank state are $P_{3}$, $T_{2}$, $\rho_{3}$, $m_{0}+m_{w}\cdot q$, where $P_{3}$ is the maximum steam pressure inside the vascular bundles of turmeric chips. The vascular bundles have a steam density of $\rho_{3}$. q is the vaporization ratio of heated water. The mass of steam from the evaporation of water inside the fresh turmeric chips and the amount of steam inside the explosion reactor tank are $m_{0}+m_{w}\cdot q$. The superheated steam from the reactor tank and the evaporated fresh turmeric chips are combined within the vascular bundles of the chips, during the steam explosion process, causing a sudden change in ambient pressure and steam pressure greater than that of the reactor tank. It breaks down under steam pressure. Under the pressure of steam, fresh turmeric chips are decomposed. When the brittle fracture process of turmeric is seen as a quasi-static process, then $P_{3}\gg P_{1}\gg P_{a}$. As a result, the maximum internal steam pressure in the vascular bundles of the turmeric $P_{3}$ determines the brittle breaking.

Fresh turmeric chips were pretreated with a steam explosion process, which led to dissipated energy per mass Ems during the brittle fracture process [33].

(1) $$E=\frac{{P_{3}}^{2}-{P_{a}}^{2}}{2K_{s}\rho_{s}}m_{s}$$

(2) $$P_{3}=P_{1}+\frac{0.2\times m_{w}c_{m}{T_{1}}^{2}R_{u}}{rMV}$$

Substituting $P_{3}$ from Equation (2) into Equation (1) results in

(3) Ems=P1+0.2×mwcmT12Ru/rMV2−Pa22Ksρs

where $K_{s}$ is the turmeric bulk modulus of 576.132 MPa, which is determined by calculating the relationship between Young’s modulus in the radial direction and Poisson’s ratio in the longitudinal and tangential directions of wood at a moisture of 12% MC [34]; $\rho_{s}$ denotes the density of turmeric at a moisture level of 7.52% MC of 1049.38 kg/m^3^; $c_{m}$ denotes the specific heat value of water 4.184 kJ/kg⋅K; r denotes the heat of water vaporization at $T_{a}$ of 2425.1 kJ/kg; V denotes the volume of the reactor tank of 1.5 × 10^−3^ m^3^; M denotes the molar mass of the gas 28.97 kg/kmol; $R_{u}$ denotes the universal gas constant 8.314 kJ/kmol⋅K; $m_{w}$ denotes the mass of water in fresh turmeric chips of 87.13 g and $m_{s}$ is the mass of dried turmeric chips of 12.87 g.

#### 2.3.3. Experimental Steam Explosion Pretreatment

Experimental steam explosions were performed using 100 g of fresh turmeric chips at pressure ranging from 10 to 25 barG, temperature from 195 to 240 °C, and 4 min residence period. The dissipated energy per mass was increased according to an increase in the pressure and temperature of the steam, as indicated in Table 1. The dissipated energy per mass was linearly related to the temperature and pressure of the steam, according to the severity factor, a common term used in a steam explosion pretreatment [35].

### 2.4. Measuring the Particle Size by Laser Particle Size Distribution Analyzer (PSD)

The particle size and distribution of turmeric particles after the steam explosion and grinding process were measured with a laser particle size distribution analyzer (PSD, Mastersizer 3000, Malvern Panalytical Ltd., Worcestershire, UK). The size and distribution of turmeric particles following a steam explosion were measured in the dissipated energy per mass, ranging from 4.144 to 13.002 J/kg. For every sample, three runs of each test were conducted.

### 2.5. Process of Curcumin Extraction

**Figure 4:**
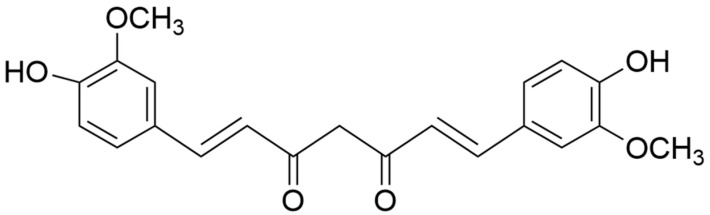
Chemical structure of curcumin.

The chemical formula of curcumin is C_21_H_20_O_6_, as shown in Figure 4, with a molecular mass of 368.4, a melting point of 183 °C, CAS No. 458-37-7, a boiling point of 418.73 °C, and a flash point of 208.9 ± 23.6 °C.

#### 2.5.1. Preparation of a Calibration Curve

Standard curcumin 20 mg (Lot # LRAD3716 purity 99.6%) was weighed and dissolved in 20 mL of 99.9% ethanol to prepare the standard solution. The standard solutions of 5, 10, 50, and 100 μL were diluted by ethanol until the volume was 10 mL, resulting in concentrations of 0.5, 1, 5, and 10 mg/L, respectively. The high-performance liquid chromatography (HPLC: Nexera LC-40) technique was used for all absorbance measurements. The column used had Luna C18(2) dimensions 250 × 4.6 mm. The solvent system used 10% acetic acid and acetonitrile in a ratio of 59:41. The detector type was UV at a wavelength of 425 nm. A calibration curve was constructed using the measured absorbance value (y-axis) and the curcumin solution concentration value (x-axis) in mg/L.

#### 2.5.2. Extraction of Curcumin from Dried Turmeric Chips, Finely Ground Turmeric, and Dried Turmeric Powder from Steam Explosion Pretreatment

**Figure 5:**
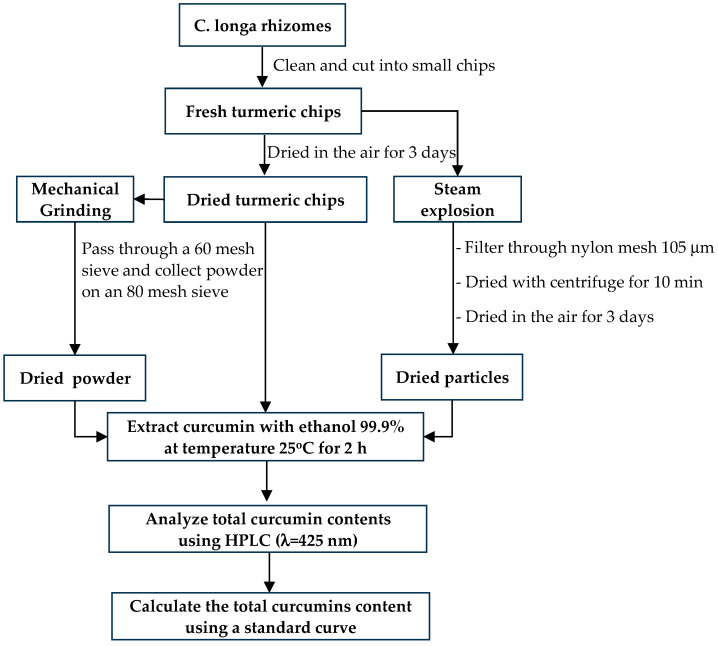
Process for extraction and determination of curcumin content from dried turmeric chips, finely ground turmeric, and dried turmeric powder from steam explosion pretreatment.

A sample solution was prepared by dissolving 3 g of dried turmeric chips in 100 mL of 99.9% ethanol. The sample was collected in an amber glass bottle, and the container was sealed and shaken. Turmeric powder finely milled with 80 mesh and turmeric powder after steam explosion at 10 barG, 15 barG, 20 barG, and 25 barG, respectively, were used to create the sample solution by dissolving 0.3 g in 10 mL of 99.9% ethanol. The amber glass bottles were kept at room temperature, and the sample solutions were taken at 2 h from 1–6 bottles, respectively. Each time collection sample had to be filtered with Whatman filter paper number 1. For 10 min, the solution was spun at 1000 rpm in a centrifuge. Subsequently, a micropipette was used to pipette 0.2 mL of the sample from each experimental set into a 15 mL amber glass bottle. Next, the compound diluted with 5 mL of 99.9% ethanol was collected in an amber glass bottle and the lid was closed tightly and the bottle was shaken. Each sample was pipetted into a 10 mL volumetric flask containing 0.2 mL. The volume was then adjusted with 99.9% ethanol until it reached 10 mL. Using the HPLC under the same operating conditions as a standard curcumin assay, samples from each experimental set were examined for curcumin extraction. The absorbance of each sample was compared to the standard curve to determine the concentration of curcumin. The procedures and specificities for extracting curcumin from turmeric are depicted in Figure 5.

### 2.6. Characterization of the Turmeric Particles After Steam Explosion Pretreatment

#### 2.6.1. Functional Group Analysis

The functional groups of turmeric and curcumin were assessed using Fourier transform infrared spectroscopy (FT-IR, FT/IR-4700, JASCO International Co., Ltd., Pfungstadt, Germany). Samples (~2 mg) were ground and mixed with KBr prior to compression into pellets for analysis. FT-IR measurement was conducted in transmission mode at wavenumbers ranging from 400 to 4000 ${cm}^{-1}$ with a 64-scan rate [36].

#### 2.6.2. Thermogravimetric Analysis (TGA) and Derivative Thermogravimetry (DTG)

The thermal characteristics of turmeric and curcumin were analyzed using a thermogravimetric analyzer (TGA/DSC 3+, auto sample robot, Mettler Toledo AG, Analytical, Schwerzenbach, Switzerland). The dried samples (~8 mg) were heated from 30 °C to 600 °C at a heating rate of 10 °C/min under a nitrogen ambient of 20 mL/min [37].

#### 2.6.3. Differential Scanning Calorimetry (DSC)

The thermal analysis was performed using a differential calorimeter (DSC 823E; Mettler Toledo, OH, USA). Approximately 5–10 mg of each sample was placed in a closed aluminum pan. Differential scanning calorimetry (DSC) analysis was performed from 25 to 300 °C at a heating rate of 10 °C/min under a nitrogen ambient of 20 mL/min [38].

## 3. Results and Discussion

### 3.1. Morphological Analysis

**Figure 6:**
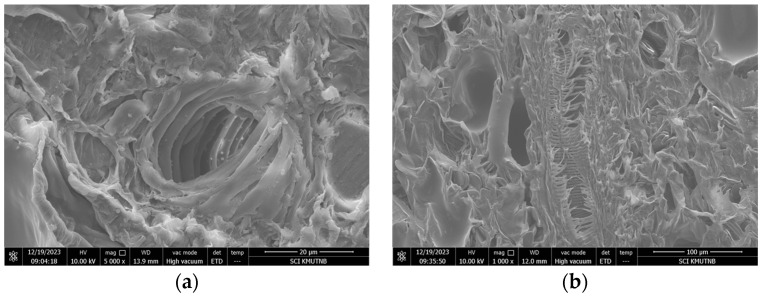
SEM of vascular bundles in transverse and longitudinal cross-section of turmeric rhizome: (**a**) transverse cross section of turmeric rhizome (5000 times magnification); (**b**) longitudinal cross section of turmeric rhizome (1000 times magnification).

The vascular bundles of turmeric are arranged transversely in the dried rhizome cross section, as seen in Figure 6. The vascular bundles have an oval cross-section shape and are coiled in layers. Additionally, the micrographs of cross and longitudinal sections show collateral vascular bundles, xylem, and phloem [39]. The high-pressure steam produced inside these cell structures facilitates the process of the steam explosion by disintegrating the cell structure.

**Figure 7:**
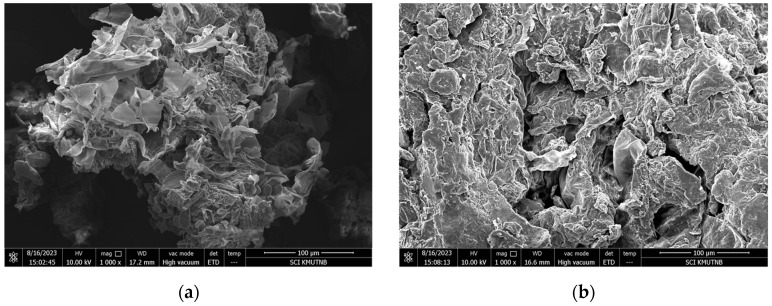
SEM of turmeric powder after grinding and after steam explosion: (**a**) after grinding (1000× magnification); (**b**) after steam explosion (1000× magnification).

The surface effects of turmeric particles were investigated using an electron microscope following the steps of fine grinding and steam explosion pretreatment, as shown in Figure 7. The 80 mesh finely ground turmeric particles at 1000× magnification had a plate surface due to the compression press of the fine grinder. A 1000× micrograph of turmeric particles after the steam explosion at 240 °C and 4 min residence time showed that the surface was destroyed, and cavities and cracks appeared on the surface of the particles. Additionally, after the steam explosion pretreatment of Seabuckthom pomace had a crinkled, curly, and holey surface [29]. However, the steam explosion pretreatment clearly elevates the porosity [40]. As a result, when ethanol penetrates and diffuses into the cell structure, the amount of extracted curcumin using the steam explosion process is greater than that from finely ground turmeric particles with the same particle size. Meanwhile, adzuki beans treated with steam explosion were shown to have bigger cell gaps and cavities, which enhanced their antioxidant activity and allowed polyphenols to be released [41].

### 3.2. Turmeric Particles Size Distribution After Steam Explosion

**Figure 8:**
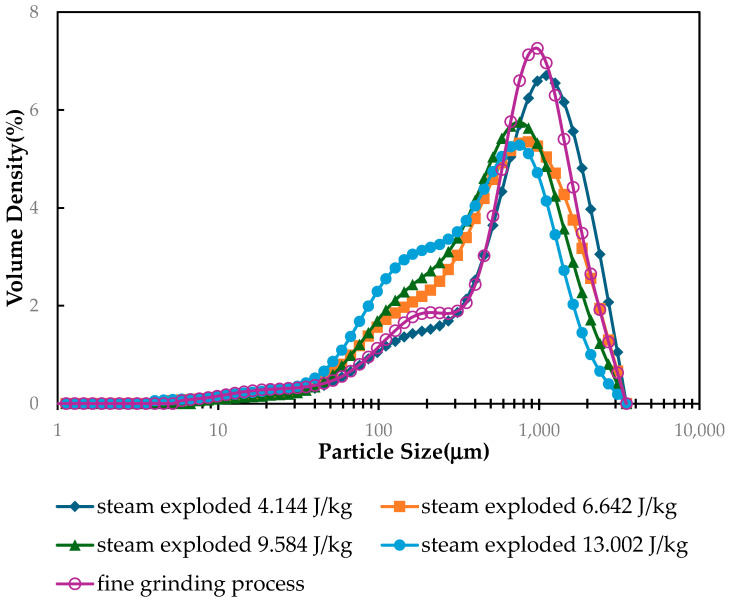
Comparison of the size distribution of fresh turmeric chips after steam explosion pretreatment with different dissipated energy per mass under 4 min residence time and fine grinding process.

The size and distribution of turmeric particles following a steam explosion were measured in the dissipated energy per mass, ranging from 4.144 to 13.002 J/kg. In the case of low dissipated energy per mass, the percentage volume density of large particles is greater than that of small particles. As dissipated energy per mass increases, the percentage volume density of small particles increases, and the percentage volume density of large particles decreases. The curve deviated in the direction of proportionately decreasing particle sizes due to the steam explosion of fresh turmeric chips with increasing dissipated energy per mass. Furthermore, when the steam pressure increased, the particle size of rice straw decreased due to the steam explosion [42]. Figure 8 shows that fresh turmeric small-sized peaks of the graph in the particle region arise as the dissipated energy per mass increases. However, at low dissipated energy per mass, fine grinding produced smaller turmeric particles than steam explosion, and at greater dissipated energy per mass, the particle size distribution was larger than that of steam explosion.

**Figure 9:**
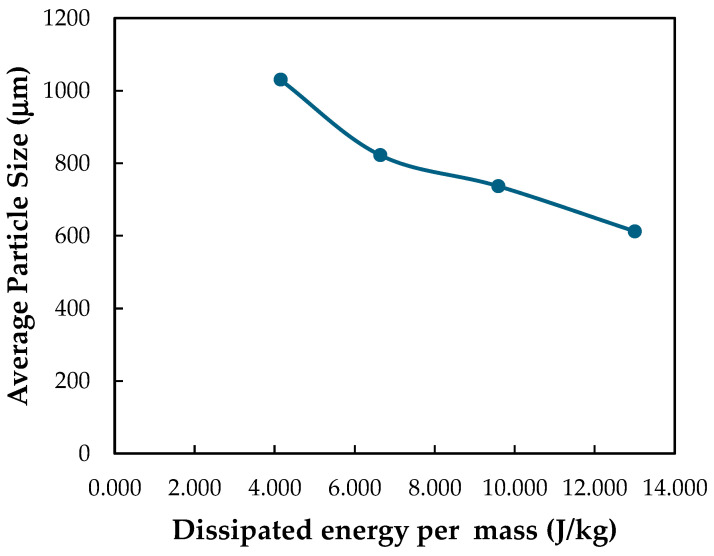
Effect of average particle size on dissipated energy per mass of turmeric fresh instantaneous decompression stage after steam explosion pretreatment.

When fresh turmeric chips undergo the steam explosion process, the absorbed steam energy from the explosion reactor tank is converted into dissipated energy per mass. Consequently, Figure 9 illustrates that the average turmeric particle size following the steam explosion was lower with the rise in dissipated energy per mass. Meanwhile, increasing the pressure difference of the explosion leads to more defibration, smaller particle size, and improving digestibility by up to 90% compared to a steam pretreatment without explosion [35].

### 3.3. The Curcumin Analysis Results by HPLC

#### 3.3.1. Calibration Curve of Curcumin Standard Results

A standard curve was created using the HPLC data for the curcumin product concentration. The concentration range of curcumin is 0–10 mg/L. Equation (4) as a function of peak area was obtained from the standard curve, which was used to calculate the concentration of curcumin as extracted from turmeric. However, the peak area of the calibration curve shows a high correlation coefficient value that is consistent with the previous studies [43].

(4) $$Y=109,587X;R^{2}=0.9999$$

where Y is the maximum peak area (mAU) and X is the curcumin concentration (mg/L).

#### 3.3.2. Analysis of Curcumin from Turmeric Chips, Finely Ground Turmeric, and Dried Turmeric Powder from Steam Explosion Pretreatment

Equation (5) provides the formula for determining the curcumin-extraction yield.

(5) $$M_{Y}(wt\%)=\frac{m_{cur}}{m_{tur}}\times 100$$

where $m_{cur}$ is mass of extractable curcumin and $m_{tur}$ is the mass of the turmeric; both are in mg units. The lowest extractable curcumin content was found in the dried turmeric chips. The maximum amount of extracted curcumin was found when the turmeric chips were finely ground to 80 mesh size. The extracted curcumin yield from turmeric particles after steam explosion was increased following the energy absorption from steam. The steam explosion process results in the highest amount of dissipated energy per mass upon maximum absorption of energy. As a result, the yield of extracted curcumin was comparable to that of finely ground turmeric. The steam explosion-generated turmeric particles exhibited surface cracks and cavities compared to the finely ground turmeric particles. This led to greater extraction of curcumin during the dissolution process with 99.9% ethanol, utilizing less energy from steam than fine grinding, resulting in 74% energy saving, as indicated in Table 2. Therefore, the steam explosion process is an optimal process when compared with untreated and mechanical grinding processes. However, the conventional mechanical methods require roughly 70% more energy to achieve the same size reduction as explosive depressurization [27].

**Figure 10:**
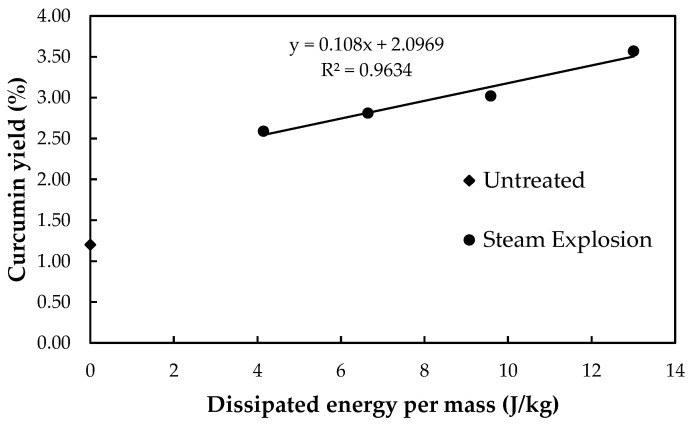
The relationship between experimental and calculated curve of curcumin yield.

The relationship between the dissipated energy per mass and the curcumin yield of the untreated and steam explosion processes from the experimental results is depicted in Figure 10. Equation (6) is a good fit on the linear correlation coefficient $R^{2}=0.9634$ for the dissipated energy per mass in the range of 4.144 to 13.002 J/kg. It was created to estimate the curcumin yield based on the energy dissipated per mass, as indicated in Table 2. The predicted results were error less than 5%. Figure 9 shows that after the steam explosion process, the average particle size tends to decrease without changing. Figure 10 shows that dissipated energy per mass increasing tends to increase curcumin yield. However, the structure of turmeric particles after the steam explosion process is one of the variables affecting the curcumin yield in addition to the variables of temperature, particle size, mixing time, and solvent (ethanol) to meal ratio used to consider the optimal conditions for curcumin extraction [44]. In comparison, the excessive steam explosion of grape pomace decreased the yield of bound phenolics and flavonoids [45].

(6) $$Y=0.108\left(\frac{E}{m_{s}}\right)+2.0969$$

### 3.4. Characteristics of the Turmeric Particles After Steam Explosion Pretreatment

#### 3.4.1. Effect of Functional Group of Curcumin and Turmeric by FTIR

**Figure 11:**
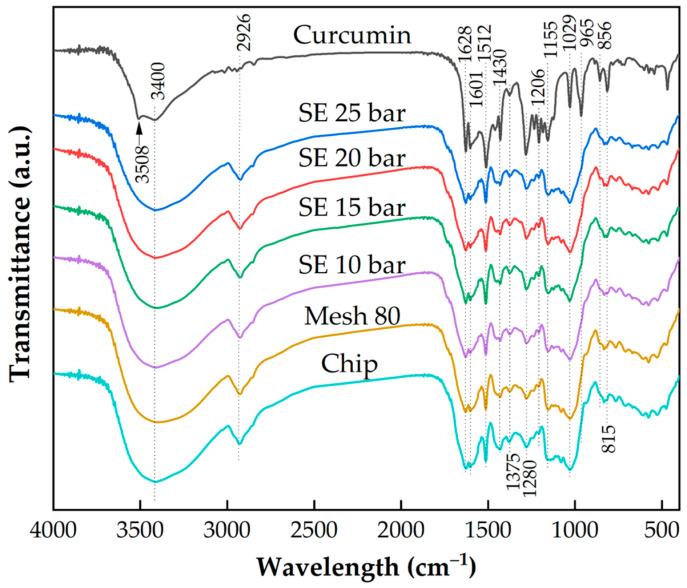
FTIR spectra of curcumin product, turmeric particles after steam explosion, grinding 80 mesh, and dried chips.

The results of FTIR analysis comparing curcumin, dried turmeric chips, grinding, and steam explosion process with steam pressure of 10 barG to 25 barG are shown in Figure 11. The chemical structure does not change, which shows that turmeric in the steam explosion process does not affect the decomposition of the compounds within turmeric. In addition, the chemical structure remains the same as that of the curcumin product, which confirms that there are curcumin components in dried turmeric. A comparison of the structural and chemical compositions of the natural and steam explosion-treated samples revealed no differences in the effect of steam explosion on the structural composition of camellia seed cake protein [46].

In the curcumin product and turmeric particle samples, there are sharp peaks in 3400 ${cm}^{-1}$, indicating that the samples have strong phenolic O-H stretching. These peaks at 2926 ${cm}^{-1}$ indicate a C-H methyl ring. These peaks are not characteristic for curcumin, which confirmed the existence of other compounds in turmeric particles. However, the peaks decreased in curcumin, which is consistent with previous studies [47]. A peak at 3508 ${cm}^{-1}$ was detected in the curcumin product only, indicating that the product has O-H stretching of the phenol group, intra-molecular H-bound [48]. In addition, there are also several peaks in spectra found in curcumin that decrease in turmeric spectra, such as at 1029 and 1280 ${cm}^{-1}$. The extraction process made these compounds increase. Detailed information about the infrared absorptions observed for various bonded groups is shown in Table 3.

#### 3.4.2. Effect of Thermal Decomposition Properties of Curcumin and Turmeric by TG and DTG

**Figure 12:**
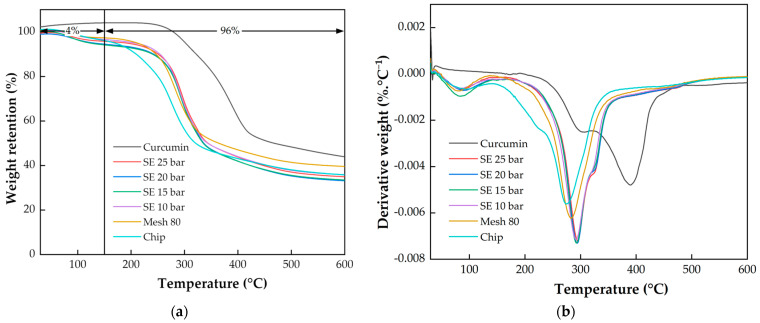
TG/DTG curves of curcumin and turmeric at different processes: (**a**) TG; (**b**) DTG.

Figure 12 shows the TG/DTG curves for curcumin and turmeric at different processes where the components inside, such as starch and sugar, affect the thermal properties [52]. However, the thermal characteristics from the steam explosion process have not changed. As can be seen from Figure 12a, the mass loss of turmeric starts at about 50 °C and ends at about 600 °C. The thermal decomposition of turmeric below 600 °C occurs in two stages. There is an inflection point at about 150 °C in the DTG curve as shown in Figure 12b, which can be regarded as the endpoint of stage I. Therefore, stage I, whose mass loss is 4%, starts at about 50 °C and ends at about 150 °C, and is characterized by a DTG peak at about 75 °C for turmeric at different processes. However, these peaks were due to moisture loss during the drying step [53,54]. Stage II, whose mass loss is 96%, begins at about 150 °C and ends at about 600 °C, which involves a DTG peak at 391.15 °C for the curcumin product, at 296.09 °C to 299.18 °C for the steam explosion process with pressure ranging from 10 barG to 25 barG, at 286.85 °C for the grinding process, and at 278.47 °C for chips. However, the peak shift of the graph in Figure 12b shows that the compounds of biomass are different [53,54].

Because the curcumin product particles were pure compounds, they had the highest decomposition temperature and residual percentage. As compared to the finely ground turmeric and the turmeric chips, the turmeric particles after the steam explosion process had a high decomposition temperature and a low percentage residual. This suggests that the turmeric passed the steam explosion process and contained more pure compounds, less cellulose, and less water-soluble compounds, as shown in Table 4.

#### 3.4.3. Effect of Thermal Properties of Curcumin and Turmeric by DSC

**Figure 13:**
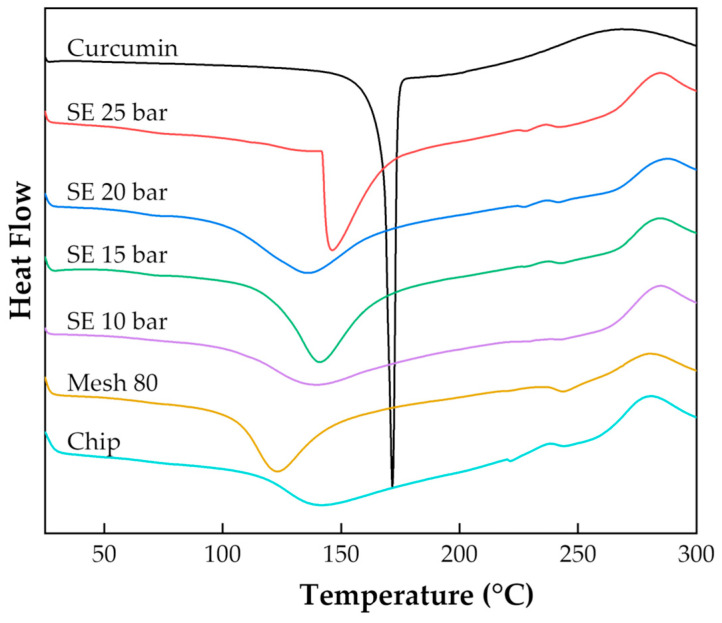
DSC thermogram of pure curcumin and turmeric at different process.

The thermal behavior of curcumin and turmeric was evaluated at temperatures ranging from 25 to 300 °C with a heating rate of 10 °C/min. Figure 13 shows the DSC thermogram of curcumin; the melting temperature ($T_{m}$) and melting enthalpy (${∆H}_{m}$) of curcumin are 170 °C and 115.28 J/g, respectively. However, the measured $T_{m}$ value was near previous studies [55].

Turmeric particles after the steam explosion process have a higher melting temperature than finely ground turmeric because the compounds have more purity with detail as shown in Table 5. More purity compounds are produced from turmeric particles that go through a high-pressure steam explosion process.

## 4. Conclusions

Steam explosion is an environmentally benign process and requires less energy than mechanical processes. With steam explosion on fresh turmeric chips, the chips disintegrated under the influence of dissipated energy per mass, with an average particle size decreasing as dissipated energy per mass increased. A high yield of curcumin extraction was obtained by the steam explosion technique, which disintegrated into small particles with increasing dissipated energy per mass where the particle surface was cracked and there were cavities. This resulted in a curcumin yield of 3.24%, compared to the 3.98% obtained from the grinding process. The steam explosion of fresh turmeric chips saves 74% of the energy compared to the fine grinding process. A linear correlation was used to estimate the curcumin yield from turmeric powder after the steam explosion process that was similar to the experimental results. Concerning the turmeric compounds after the steam explosion process, it was found that there was no change in the chemical structure, higher thermal decomposition properties, and higher purity. When compared to the fine grinding method, the steam explosion process was found to be more appropriate for extracting curcumin when applied to fresh turmeric chips. In terms of commercial application, it is appropriate to pretreat herbal plants before extraction in order to produce more compounds. This is especially useful for continuous process development, which aims to enhance yield and reduce production cost in future studies.

## Figures and Tables

**Table 1:** The experimental conditions for steam explosion of fresh turmeric chips.

Steam Pressure P_1_ (barG)	Steam Temperature T_1_ (°C)	Dissipated Energy per Mass Ems (J/kg)
10	195	4.144
15	214	6.642
20	228	9.584
25	240	13.002

**Table 2:** Curcumin yields in experimental conditions.

Turmeric Samples	Dissipated Energy per Mass Ems (J/kg)	Particle Size Avg. (μm)	Total Energy per Mass (kJ/kg)	Energy Change (%)	Curcumin Yield (%)		
					Exp	Predicted	% Error
Chips	-	10,000	-	-	1.20	-	-
Powder 80 mesh	-	250	1952	-	3.98	-	-
Powder SE 10 barG	4.144	1030	210	89	2.25	2.21	1.78
Powder SE 15 barG	6.642	822	312	84	2.48	2.48	0.00
Powder SE 20 barG	9.584	737	413	79	2.71	2.81	3.69
Powder SE 25 barG	13.002	612	515	74	3.24	3.18	1.85

**Table 3:** Functional group of curcumin in FTIR.

Functional Group	Wavenumber (from Experiment, cm^−1^)	Wavenumber (from Reference, cm^−1^)	References
Phenolic O-H stretching	3400	3426	[47]
C=C vibrations	1512	1510, 1544	[47,49]
Olefinic C-H bending vibration	1430	1439, 1405	[47,49]
Aromatic C-O stretching vibration	1280	1277, 1285	[47,50]
C-O-C stretching vibration	1029	1034, 1027	[47,51]
C-H methyl ring	2926	2924	[47]

**Table 4:** Thermal decomposition properties of curcumin and turmeric by TG/DTG.

Samples	T_max_ (°C)	Residue (%)
Curcumin	391.15	42.30
Powder SE 25 barG	299.18	34.42
Powder SE 20 barG	298.07	32.61
Powder SE 15 barG	296.73	32.96
Powder SE 10 barG	296.09	35.64
Powder 80 mesh	286.85	38.99
Chips	278.47	35.06

**Table 5:** Thermal properties of curcumin and turmeric by DSC.

Samples	T_m_ (°C)	$∆H_{m}$ (J/g)
Curcumin	170.00	115.28
Powder SE 25 barG	145.75	72.37
Powder SE 20 barG	135.70	168.70
Powder SE 15 barG	140.60	171.81
Powder SE 10 barG	139.73	131.77
Powder 80 mesh	122.86	167.52
Chips	141.85	104.25

## Data Availability

All the data are available within this manuscript.
